# Construction of interactive health education model for adolescents based on affective computing

**DOI:** 10.3389/fpsyg.2022.970513

**Published:** 2022-08-11

**Authors:** Xieping Chen, Yu Zhang, Qian Xie

**Affiliations:** ^1^School of Educational Science, Leshan Normal University, Leshan, China; ^2^College of Teachers, Chengdu University, Chengdu, China

**Keywords:** health education model, affective computing, ICABoost algorithm, interactive emotional algorithm, youth health education

## Abstract

At present, people mainly focus on health education for adolescents. The health education of adolescents is related to future of adolescents. In youth, their emotions are easily influenced. Therefore, this manuscript constructs an interactive health education model for adolescents through affective computing. Researchers in various countries have done a lot of research on human–computer interaction, and affective computing is one of the research hotspots. This manuscript aims to study the use of affective computing to construct an interactive health education model for adolescents. It proposed an interactive emotional algorithm based on emotional computing and focuses on the ICABoost algorithm. The experimental results of this paper show that the surveyed junior high school students are divided into three grades: the first, second, and third grades. Among them, 11, 11, and 13 were mentally healthy, with a total percentage of only 18.5%; 16, 14, and 16 were moderately severe in health education, accounting for 24.3%. The percentage of severe cases was 29.6%. It can be seen that, through the investigation of this manuscript, it can be seen that today’s youth health education should be paid attention to. Only by constructing a corresponding interactive health education model for young people can we promote the comprehensive and healthy development of young people.

## Introduction

Teenagers are in a special period of growth and learning, especially when facing the college entrance examination. The pressures of parents, schools, and society create challenges for the healthy growth of students. In this case, psychology must participate directly in the school’s mental health education. Psychological knowledge needs to be translated into applied form. It is essential to directly contribute to the health education of young people and promote the healthy growth of students’ mental health. In the practice of mental health education, school management is not only institutional management but also more importantly, humanized management. The focus of management is people-oriented. Mental health education is very important to the growth of students. Mental health education truly embodies the modern high-quality education concept of “student-centered, student-oriented development.” It is necessary to insist on facing the whole, combine health education with morality, intelligence, physique, beauty, and labor, and integrate it into the whole process of education, teaching, management, and service. It is necessary to give full play to the roles of school teachers and full-time and part-time school doctors to build a health education system that is oriented to everyone and everyone is responsible for.

In recent years, the state believes that improving the psychological quality of young people is an important part of implementing high-quality education. As China’s economy grows rapidly in society and the continuous deepening of the reform of China’s education system, the psychological pressure on young people has increased, and the voice for improving the health education of young people is getting louder and louder. Adolescents are a group that society and parents place a lot of expectations on. Health education not only means that teenagers are free from physical diseases but also means that they are positive and healthy psychologically, and they will not retreat in the face of setbacks.

The innovation of this manuscript: (1) introduces the relevant theoretical knowledge of health education and affective computing and proposes an emotional interaction algorithm based on emotional computing and analyzes how the emotional interaction algorithm plays a role in the construction of an interactive health education model for adolescents. (2) This manuscript conducts an experimental comparative analysis of adolescents who have received the interactive health education model. Through comparison, it is concluded that the interactive health education model is conducive to the smooth progress of adolescent health education and can improve the quality of health education.

## Related work

With the comprehensive development of young people, the state has begun to pay more and more attention to the health education of young people. Babatunde E O found that health education and a healthy lifestyle can have a positive impact on adolescent academic performance. Learning experiences can help students accurately assess the level of risk-taking behavior of their peers. He emphasized the value of health, and insisting on healthy attitudes and beliefs is the most important. His research used multiple regression descriptive and inferential statistics to analyze the data (Babatunde, 2017). Moran M. B. found that several recent health behavioral interventions were directed at high-risk cohorts. To establish a scientific basis for using this approach, he positioned the approach as a cultural strategy for healthy behavioral interventions for adolescents and critically evaluated the benefits and limitations of this approach (Moran et al., 2017). Odusola A. O. discovered a free mobile phone app that contained comprehensive health information and determined that the app had the greatest appeal among teens. He surveyed the teens. Participant responses to health questionnaires, interviews, and time spent viewing the app were used to determine the feasibility of the protocol (Odusola et al., 2017). Kabasakal E. found that preventable diseases constitute a serious problem worldwide, and the role of primary care professionals in promoting health is particularly important. He surveyed medical professionals and found that 33.3% of healthcare professionals plan to receive health education, and about half of them actively practice health education and health promotion skills (Kabasakal and Kublay, 2017). Delany C. found that in higher education, assessment is key to student learning and assessment promotes that continuous learning is highly valued. However, assessment tasks to achieve these types of thinking skills and take action in professional practice have received little attention. He used constructivist qualitative methods to explore learning objectives and assessment strategies used in health higher education (Delany et al., 2017). Weaver S. found that lifestyle-related health issues are a significant problem in other countries. In free clinics, health education programs have the potential to reduce the incidence of health problems among vulnerable populations. He used direct observations based on the theory of planned behavior (TPB) to describe health education programs in free clinics serving vulnerable populations. The primary data source was field records of observations of free clinic health education courses. But he lacked the sense of responsibility or willpower required to engage in health-related behaviors (Weaver et al., 2017). Scholars have found that the importance of health education for young people is getting higher and higher, but the traditional health education model can no longer meet the needs of society.

Affective computing is an important step in the intelligent integration of machines and humans, and it is also an important part of the field of human–computer interaction. Enabling machines to understand people’s emotions could greatly reduce the workload for people in a variety of industries. This greatly improves the efficiency and experience, and at the same time, it enables them to better understand themselves and improve their quality of life. Zhou Q. proposed a multi-layer emotional decision-making model by establishing the mapping relationship between characters, emotions, and actions. This model reflects the changes in emotions and emotional spaces based on different roles and provides a reference for the modeling of human–computer interaction systems (Zhou, 2018). Xin found that the emerging field of affective computing focuses on enhancing the ability of computers to understand and appropriately respond to people’s emotional states in human–computer interaction, and shows great potential for wide-ranging applications. He suggested that researchers should be fully aware of the limitations of commonly used emotion models and be cautious about widely accepted assumptions about the EEG-emotion relationship (Xin, 2019). Maria Garcia-Garcia J.’s discovery of the relevance of psychophysiological measures to affective computing and sentiment analysis applications is widely recognized. He studied the information content of parameters derived from cardiovascular and other modalities and proposed methods for emotional feature extraction (Maria et al., 2018). In the interactive health education model for teenagers, it can use affective computing very well to identify the emotions of teenagers, so as to find out whether the health status of teenagers is healthy or not. Based on the theory of emotional psychology, it analyzed the factors that affect emotional states and their changes.

## Emotional interaction algorithm based on emotional computing

### Development of adolescent health education

**FIGURE 1:**
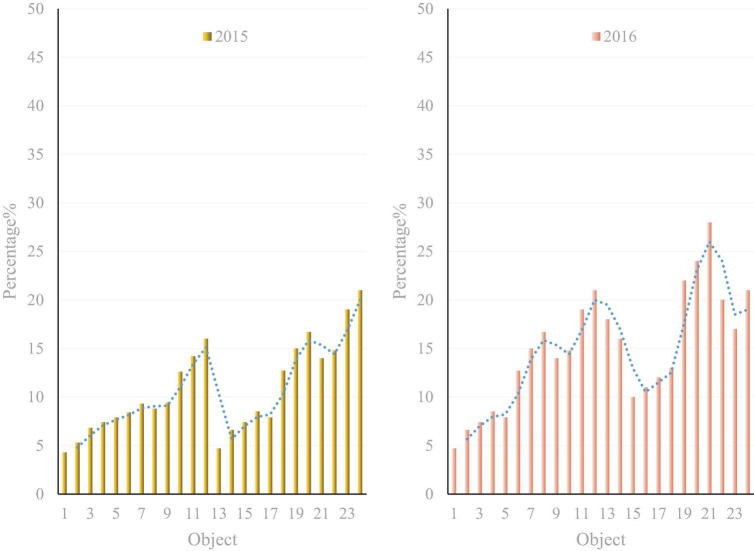
The development scale of adolescent health education in recent years.

Health education and health promotion are the precursors to primary healthcare. Health education is the key to fulfilling the mission of primary healthcare. Health education has an important position and value in achieving all health goals, social goals, and economic goals. Adolescents are in a period of rapid physical and mental change. They are influenced by factors such as social, natural environment, school, and family changes in their pursuit of self-identity, life goals, and meaning in life. In recent years, adolescents cannot bear setbacks due to psychological pressure, have low resistance to pressure, and lack enthusiasm to actively solve problems (Sim et al., 2017). However, some young people who have experienced crises and stressful situations can overcome difficulties and setbacks and develop excellent mental health characteristics and abilities compared to other youths who are more likely to fall into difficulties and depression. The development scale of adolescent health education in recent years is shown in Figure 1.

As shown in Figure 1, after the development of the country, the development scale of health research has become quite large. People are more concerned about their health than in the past. Therefore, everyone should know the necessary health knowledge. Health education in other countries has the following characteristics: Each school has specialists in health education. Health education has developed from school to society and family. Government agencies attach great importance to health education in mental health education. That is, it puts the focus on the development and growth of youth. The health education work has gradually matured and entered the stage of continuous improvement (Jain et al., 2022).

### Status quo of affective computing research

Affective computing aims to make computers more intelligent by giving them the ability to recognize, understand and express human emotions. Affective computing makes people’s lives more intelligent, which is the main goal of research in the field of artificial intelligence (AI) in recent years. Among them, human–computer interaction is the current research hotspot in the field of AI. Affective computing is one of the important manifestations of natural human–computer interaction. It analyzes and recognizes changes in signals such as physiology, posture, expression, and tone caused by changes in human emotions through systems such as sensors, video, and audio. This in turn understands human emotions and gives accurate and appropriate responses (Pickett et al., 2017).

The computer analyzes and processes the signals collected from the sensor to obtain the emotional state of the other party (person), which is called emotion recognition. In emotion recognition, the modalities contained in emotion mainly include facial expressions, behavioral gestures, speech, and physiological signals. Although faces, gestures, and voices can express certain emotions independently, people always express these information comprehensively in the process of mutual communication, such as smiling and dancing when they are happy and wide-eyed when they are angry.

Emotion recognition is one of the most basic and important research contents in the field of emotion computing. In order to achieve a harmonious and efficient interaction environment between humans and computers, computers have higher and more comprehensive intelligence.

### Affective computing based on linear discriminant model

Since the ultimate purpose of the entire affective computing model is to be able to discriminate the selected effective features, proper classification model selection is very necessary (Adem et al., 2022). There are many kinds of classification models, and this paper chooses LDA as the basic classifier. This is because LDA is a supervised model, which is more suitable for small training data such as people. Moreover, LDA assumes that the model distribution is a multidimensional Gaussian mixture model. It finds that this assumption is true through the analysis of the results. In addition, the LDA model can reduce the dimensionality of the features, making the data more visualized and more conducive to the overall analysis of the model (Babamiri et al., 2017).

LatentDirichletAllocation (LDA) is a document topic generation model, also known as a three-layer Bayesian probability model, which includes the three-layer structure of words, topics, and documents. The LDA model is a classical algorithm in statistics, which is often used in classification and supervised feature dimensionality reduction scenarios. It assumes that the sample data are normally distributed and the covariance matrix of each sample distribution is the same (Shabanian et al., 2018). In discriminant analysis, there are three scatter matrices to be considered for a dataset: intra-class scatter matrix *S*_b_, inter-class scatter matrix *S*_w_, and mixed scatter matrix *S*_*m*_. The calculation method of the intra-class scatter matrix *S*_b_ is as formula 1:


(1) $$S_{\text{b}}=\sum_{i=1}^{c}N_{i}\,\left(\mu_{\text{i}}-\overset{-}{a}\right)\,{\left(\mu_{\text{i}}-\overset{-}{a}\right)}^{T}=\Phi_{\text{b}}\,\Phi_{\text{b}}^{T}$$

The calculation method of the inter-class scatter matrix *S*_*w*_ is as formula 2:


(2) $$S_{w}=\sum_{i=1}^{\text{c}}\sum_{j\in C_{\text{i}}}\left(a_{j}-\mu_{\text{i}}\right)\,{\left(a_{j}-\mu_{\text{i}}\right)}^{T}=\Phi_{w}\,\Phi_{\text{w}}^{\text{T}}$$

The calculation method of mixed scatter matrix *S*_*m*_ is as formula 3:


(3) $$S_{m}=S_{w}+S_{\text{b}}=\sum_{i=1}^{N}\left(a_{\text{i}}-\overset{-}{a}\right)\,{\left(a_{\text{i}}-\overset{-}{a}\right)}^{T}$$

N is the total number of samples, *a*_i_ is the number of samples belonging to category *C*_i_, μ_i_ is the sampling mean of category *C*_i_, and *a* is the mean vector of all samples, *as* in formula 4:


(4) $$\mu_{\text{i}}=\frac{1}{N}\,\sum_{i\in C_{\text{i}}}a_{\text{i}}$$

The idea of LDA is to find the optimal transformation matrix W that satisfies formula 5:


(5) $$J\,\left(W\right)=\arg\max\frac{\left|W^{T}\,S_{\text{b}}\,W\right|}{\left|W^{T}\,S_{w}\,W\right|}$$

Transformation matrix is a concept in mathematical linear algebra. In linear algebra, linear transformations can be represented by matrices. Only by obtaining the transformation matrix W, the mean and variance of each category after mapping can be obtained, and the final mapping result can be obtained. Essentially, this is looking for a mapping method that maximizes the between-class differences while minimizing the intra-class gap (Mohamad et al., 2018).

The ultimate purpose of using the LDA model is to find the transformation matrix W, which can find its distribution in the mapping space by mapping the new data during the testing process. It also performs type discrimination based on the mean and variance results obtained during training. The method is to compare the probabilities under different categories and select the category with the highest probability of the data category as the category represented by the new data. Its probability calculation for new data is as formula 6:


(6) $$P\left(b=c|a,\mu_{c},\sum \right)\infty \exp\left[\mu_{c}^{T}\sum^{-1}a-\frac{1}{2}\mu_{c}^{T}\right]$$

μ_*c*_ is the mean of category c, ∑ is the covariance matrix, *a* is the input data, and T is the judgment category.

### AdaBoost expression recognition algorithm

#### Expression independent component analysis

**FIGURE 2:**
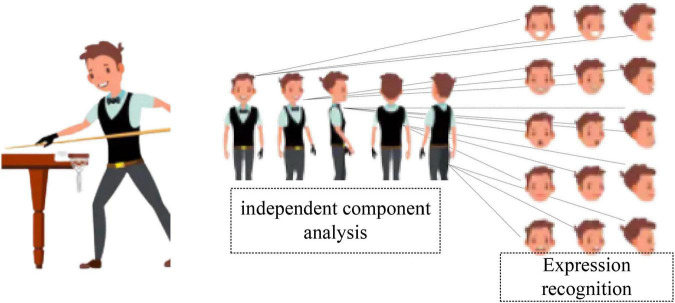
Expression of independent component analysis.

Independent component analysis (ICA) is a new signal processing technique developed in the 1990s. The main purpose of the independent component analysis is to find a linear transformation for non-Gaussian data. It makes the output components to be statistically independent or as independent as possible, so the criterion for judging independence is very critical. The expression independent component analysis is shown in Figure 2.

As shown in Figure 2, mathematically independent and non-Gaussian are equivalent. It mainly has two judgment criteria: negentropy and kurtosis. However, the kurtosis criterion is sensitive to the external conditions of the data and is unstable. Therefore, negative entropy is generally used to determine whether two variables are independent. Negative entropy is the decrease of entropy, which is the negative change in entropy function. Negative entropy is a measure of the ordered, organized, and complex state of a material system.

For the mixed signal, its probability density is closer to the Gaussian distribution than the probability distribution of any one source signal. Conversely, maximizing the non-Gaussianness of the signal is consistent with maximizing the statistical independence of the signal, which is just the basic principle of ICA. An approximate formula can be used to measure the non-Gaussianness such as formula 7:


(7) $$J_{G}\,\left(b\right)\approx \sum_{i=1}^{p}k_{\text{i}}\,{\left[E\,\left\{G_{\text{i}}\,\left(b\right)\right\}-\left\{G_{\text{i}}\,\left(v\right)\right\}\right]}^{2}$$

*k*_i_ is some positive constant, *v* is a Gaussian variable with zero mean and unit variance, and function *G*_i_ is a non-quadratic function.

It assumes that a has undergone centralization and pre-whitening processing, and the maximum value *J*_*G*_(*b*) is obtained at an optimal solution, and the optimal solution of *J*_*G*_(*b*) satisfies Formula 8:


(8) $$E\,\left\{x\,g\,w^{\text{t}}\,x\right\}-\beta \,w=0$$

β is a constant, and when *w* is the optimal value, formula 9 can be easily obtained:


(9) $$\beta =E\,\left\{w_{0}^{t}\,x\,g\,\left(w_{0}^{t}\,x\right)\right\}$$

According to this formula, the approximate value of β in the iteration can be calculated.

#### AdaBoost classification algorithm

The AdaBoost classification algorithm proposed in this manuscript is the core of expression recognition. Its basic idea is to organize and classify the feature components by constructing the AdaBoost weak classifier based on the ICA component of the special area of the face. The AdaBoost algorithm is done by looping. In each loop, it selects a weak classifier. The only requirement for weak classifiers is that their classification accuracy greater than 0.5. Its core idea is to combine the power of the weak estimator to predict the samples that are difficult to evaluate, thus forming a strong estimator.

In this manuscript, each independent component may constitute a weak classifier. A simple test needs to be done first through the recognition effect of their Euclidean distance. This is to find those independent components that can really constitute a weak classifier as in formula 10:


(10) $$\sum_{i=1}^{Q_{k}}f\,\left(P_{k\,\left(i\right),j}\right)≻\frac{Q_{K}}{2}$$

Then it is considered that this independent component $\frac{Q_{K}}{2}$ can constitute a classifier on the kth expression.


(11) $$f\,\left(P_{k\,\left(i\right),j}\right)=\left\{ \begin{array}{c} 1,i\,f\,P_{k\,\left(i\right),j}≻u_{j}\,\left(k\right)\\ 0,o\,t\,h\,e\,r\,w\,i\,s\,e \end{array}\right.$$

Once the M independent components are selected and all form the corresponding weak classifier, using the continuous multi-class AdaBoost algorithm, the weak classifier should be configured with a feature f and a threshold value of θ_*k*_ as shown in formula 12:


(12) $$h\,\left(f,k\right)=\left\{ \begin{array}{c} 1,i\,f\to l≻\theta_{\text{k}}\\ 0,f\to o\,t\,h\,e\,r\,w\,i\,s\,e \end{array}\right.$$

It uses B to represent the sample space and *l* to represent the label space, then a simple multi-class multi-label problem can be represented by a pair of data *B*[*l*]. Here *B* ∈ Φ, which is defined as formula 13:


(13) $$B\,\left[l\right]=\left\{ \begin{array}{c} 1,i\,f\to l\in B\\ -1,i\,f\to l\notin B \end{array}\right.$$

Then the AdaBoost classification algorithm can be defined as follows:

Under distribution condition D_t_, a weak classifier is selected from the weak classifiers, and the weights are normalized so that it satisfies a certain distribution as shown in formula 14:


(14) $$D_{t+1,i}=\frac{D_{t+1,i}}{\sum_{j=1}^{n}D_{t+1,j}}$$

The AdaBoost algorithm proposed in this manuscript fully considers the advantages of the ICA and AdaBoost algorithms. One can generate possible excellent features, and one can select those features that really have excellent discriminant analysis performance from some good and bad features.

In general, the algorithm mainly has the following characteristics: By repeatedly running the ICA algorithm with two different expressions, the true independent components with excellent performance can be obtained with a high probability. This has a huge impact on the subsequent identification stages. Compared with the traditional AdaBoost algorithm, the feature to be selected in this paper is the ICA feature. Therefore, the dimension of the feature space greatly reduced without losing a lot of information. So its execution efficiency is higher and the recognition result is more accurate.

### ECEM emotional interaction model

At present, domestic and foreign research institutions have carried out research on artificial psychology and emotional robots. Their research goal is to build an intelligent terminal with emotion, so that the machine has human-like emotion. From another perspective, this manuscript provides auxiliary measures to help human emotion control and emotion transformation based on understanding human emotion. This is of great help in stimulating learners’ enthusiasm for learning and adjusting their learning status.

**FIGURE 3:**
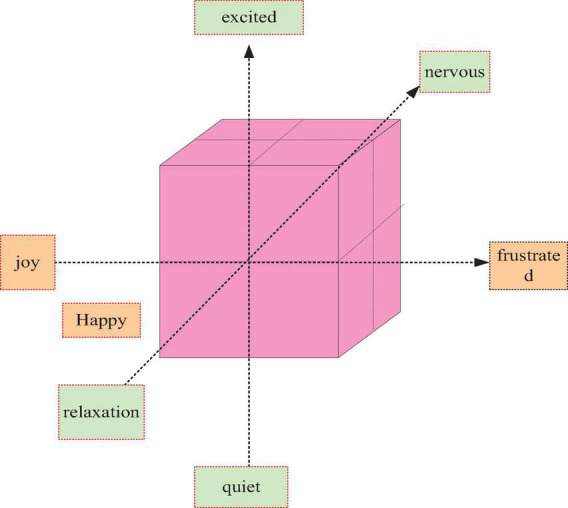
3D model of emotions.

On the basis of the three-dimensional theory of emotion, considering the possible emotional states of learners in remote virtual teaching, people have established an emotional space with four components. The ECEM emotional interaction model is shown in Figure 3.

As shown in Figure 3, it not only expands and includes emotional intelligence but also deeply reflects higher-level thinking and wisdom in human intelligence. Human emotion and cognition research has opened a new direction for computer applications. The emotion space is divided into four subspaces by three pairs of emotion elements. In order to simplify the problem, the experiment also considers the distribution of students’ emotions, which are excited, happy, frustrated, and angry. Among them, excitement and joy are regarded as positive emotions, that is, emotional states that can promote or improve students’ learning interest and efficiency. Frustration and anger are regarded as negative emotions, that is, emotional states that inhibit or reduce the efficiency of health education for students. The dots in the three-dimensional coordinate system are neutral emotions, which neither promote nor inhibit students’ health education.

It sets the number of emotional state space sets to (*i* = 1,2,…,*N*), and *X*_i_ represents the number of basic emotional states. In this manuscript, five basic emotional states are used, and the emotional state is represented by a random variable A. It sets *X*_i_ as the probability of taking the nth emotional state and satisfies formula 15:


(15) $$\sum_{i=1}^{N}X_{\text{i}}=1,0\leq X_{\text{i}}\leq 1\left(i=1,2,\ldots ,N\right)$$

In this way, the probability space model of affective states can be expressed as formula 16:


(16) $$\left(\begin{array}{c} S\\ X \end{array}\right)=\left[\begin{array}{cc} s_{1} & s_{n}\\ x_{1} & x_{n} \end{array}\right]$$

After the emotional state is determined, the transition rules between states must be defined. That is, under certain conditions, the transition probability problem needs to be transformed from the source emotional state to the target state. In addition, it also defines the problem of how to choose the optimal external incentive measures when the source state and target state are known.

Therefore, some scholars proposed an HMM model containing three emotional states. Hidden Markov Model (HMM) is a classic machine learning model, which has been widely used in language recognition, natural language processing, pattern recognition, and other fields. The following HMM parameters determined for each emotional state can be described by the following parameters as in formula 17:


(17) $$P\,\left(O|\lambda \right)=\sum_{i=1}^{\text{N}}\beta_{l}\,\left(i\right)$$

The HMM model is an iterative algorithm, and the user gives the empirical value of each parameter at the first instant. With continuous repetition, each parameter tends to gradually become a more appropriate value. As in formula 18:


(18) $$\gamma_{t}\,\left(i\right)=\sum_{j=1}^{\text{N}}\zeta_{t}\,\left(i,j\right)$$

In the absence of external stimuli, the mood will gradually fade with the passage of time, and its emotional intensity will gradually weaken. It assumes that the ideal emotional state is E, and the emotional state corresponding to the current time t is E(t), then the rate of change in the current emotional state is equal to the difference between the emotional state at the current moment and the ideal emotional state. Emotional motivation method is a management method that stimulates the enthusiasm of the managers through good emotional relationships, thereby improving efficiency. Its emotion dilution process can be described as formula 19:


(19) $$\frac{d\,E\,\left(t\right)}{d\,t}=\beta \,\left[E-E\,\left(t\right)\right]$$

β is the emotion fade factor, which reflects the speed of emotion fade.

Aiming at the possible emotional states of adolescents in the process of health education, this paper proposes an ECEM algorithm to achieve emotional monitoring and emotional stimulation of adolescents in the process of health education.

The emotional domain TEA is defined as a one-dimensional representation of the learner’s learning emotion, and its calculation is as shown in formula 20:


(20) $$t\,e\,a\,\left(\pi_{\text{i}}\right)=\pi \cdot C$$

This manuscript performs expression classification on 50 images of the database. In the laboratory, eigenfaces were assigned to each distinct face. The eigenface represents the expression of neutral emotion and is the reference frame for judging other types of expressions, which improves the accuracy of expression classification.

For images in the facial expression database, using the method proposed in this paper is faster and takes an average of about 1 s per image, which makes real-time video processing possible. At the same time, the classification accuracy of the algorithm for simple expressions can basically meet the needs of practical applications. In this manuscript, the ICA expression recognition method and the ICABoost expression recognition method mentioned in the text are compared and analyzed, and the 50 images are divided into five groups, as shown in Tables 1, 2.

**TABLE 1:** Comparison of ICA expression recognition rates.

Object	a	b	c	d	e
Excited	45%	52%	39%	58%	29%
Happy	50%	28%	34%	55%	18%
Calm	36%	31%	38%	53%	27%
Frustrated	39%	23%	22%	40%	20%
Angry	40%	48%	27%	44%	32%

**TABLE 2:** Comparison of ICABoost expression recognition rates.

Object	a	b	c	d	e
Excited	65%	73%	85%	89%	77%
Happy	64%	79%	82%	82%	80%
Calm	48%	70%	83%	81%	64%
Frustrated	61%	77%	79%	86%	69%
Angry	59%	76%	75%	78%	76%

As shown in Tables 1, 2, the expression recognition rates of the ICA and ICABoost algorithms are compared, and the results show that the recognition accuracy of the ICABoost algorithm is significantly higher than that of the ICA algorithm. The recognition accuracy of the ICABoost algorithm is generally above 60%, while the recognition accuracy of the ICA algorithm is about 30%.

**FIGURE 4:**
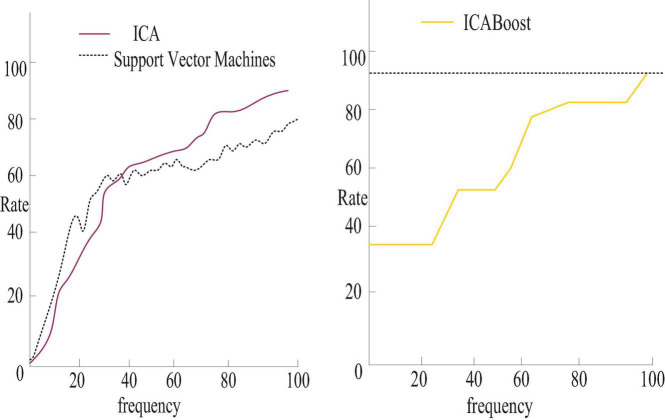
Expression recognition results of ICA and ICABoost algorithms and support vector machine methods.

At the same time, this manuscript compares the expression recognition results of ICA and ICABoost algorithms and support vector machine methods, as shown in Figure 4.

As shown in Figure 4, the ICABoost algorithm has obvious advantages over the support vector machine method. The main reason is that ICABoost makes full use of the effectiveness of ICA in feature extraction and the excellent characteristics of AdaBoosting algorithm classification in feature classification, and the algorithm effectively reduces the dimension of traditional ICA features and improves computational efficiency.

## The construction of interactive health education model–taking mental health education as an example

### The current situation of the mental health education model

#### Insufficient funds

With the attention of the national education authorities on the mental health of school students, mental health education has been paid attention to by school leaders to a certain extent, but it is far from enough. In the discussion, a scholar mentioned that due to the backwardness of the local economy and the lack of local financial revenue, the investment in education funds just meets the normal operation of the school, and the updating of equipment and the training of teachers are still far behind. The equipment is all temporary subsidies from the state and donations from social groups and individuals.

Therefore, people feel more than enough of mental health education. To ensure the development of mental health education in primary and secondary schools, the investment of funds is also crucial. The local education department should form a separate budget for mental health education, review and use it, and make it special. At the same time, it can also mobilize social volunteers, and use social funds, corporate sponsorship, and personal donations to ensure the investment of mental health education funds.

#### The school does not pay enough attention to

Regarding how to better carry out mental health education in primary and secondary schools, special institutions should be set up for management. In the discussion, the teacher of a primary school mentioned that psychological education is mostly completed by the head teacher, and the activities carried out by the head teacher are managed by the Youth League Committee. The curriculum education carried out is also managed by the age director. Mental health education has not been placed under the management and responsibility of that department and has been forgotten and ignored. To carry out mental health education, it is necessary to have a unified department for management, coordination, and cooperation. This is conducive to the development of mental health education in primary and secondary schools.

#### Insufficient publicity

The propaganda of the knowledge of mental health education among students should be strengthened, so that students can understand that it is not so shameful to say their own psychological thoughts. At the same time, it is necessary for students to master some small methods to relieve psychological pressure. People found in the interviews and discussions that many schools and teachers only use the time of class meetings to preach, which cannot achieve good results. The effect of education is superficial, and even has a serious negative impact on students. Most of the class meetings focus on students’ safety education and respect for discipline education, while ignoring students’ mental health education.

### The establishment of the interactive health education model

**FIGURE 5:**
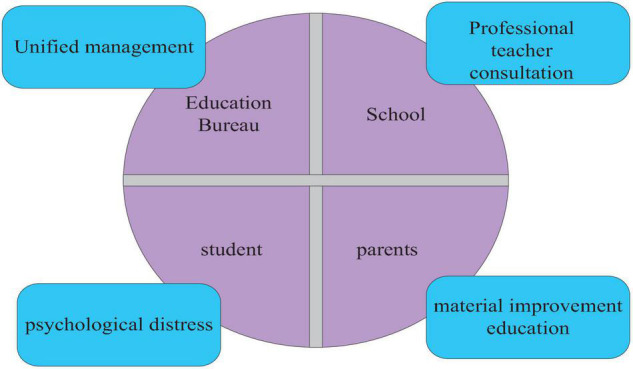
The specific content of the health education model.

The so-called interactive health education model is the model of human–computer interaction. This paper first designs the construction of the health education model from the perspective of school teachers, and then simply designs the health education model of the machine system. The health education model is to put students’ health education in a comprehensive and open environment, and it is a model that has a positive impact on students’ psychology. The specific content of the health education model is shown in Figure 5.

As shown in Figure 5, the methods of health education mainly include knowledge dissemination and behavioral intervention. The specific contents of the health education model include: giving psychological knowledge, the penetration of psychological education in the field, the creation of educational conditions, the proposition of action training, the success of extracurricular activities, the optimization of the campus environment, the implementation of psychological measurement, the implementation of psychological counseling, the improvement of family environment, social countermeasures, etc. This is very helpful for cultivating students with high psychological quality and forming a sound personality, which is also the general requirement of the new education curriculum reform.

The goals to be achieved by this pattern have the following three levels:

The first level is comprehensive and developmental. Mental health education is aimed at all students, and the main purpose is to improve the psychological quality of all students, so that they have certain methods and abilities to solve the psychological contradictions and problems in learning.

The second level is dredging and preventive. In response to possible problems or signs of students, the head teacher should adopt a guiding method rather than a strict control method. It is necessary to carry out systematic mental health education in a planned and step-by-step manner, cultivate self-psychological adjustment ability, prevent the occurrence of students’ psychological problems, and have a correct outlook on life and values.

The third level is targeted and therapeutic. The head teacher discovers and corrects the psychological problems of the students in time, makes use of the special role and advantages of the head teacher, helps them analyze the cause of the disease, finds out the method to solve the problem, and conducts treatment from learning to activities and other aspects. This eliminates their psychological barriers and helps them carry out psychological rehabilitation training.

The main feature of this model is the function and role of the heart management department. The mental health management department is established on the basis of the school’s psychological counseling room, and the executive vice-principal serves as the director of the management department, coordinating all aspects of the school to carry out the mental health education of students. Its main tasks include: formulating the school’s mental health education plan, compiling school-based teaching materials, training class teachers and teachers, undertaking mental health education courses, and providing group and individual psychological counseling to students.

### EIMBER system interactive model

**FIGURE 6:**
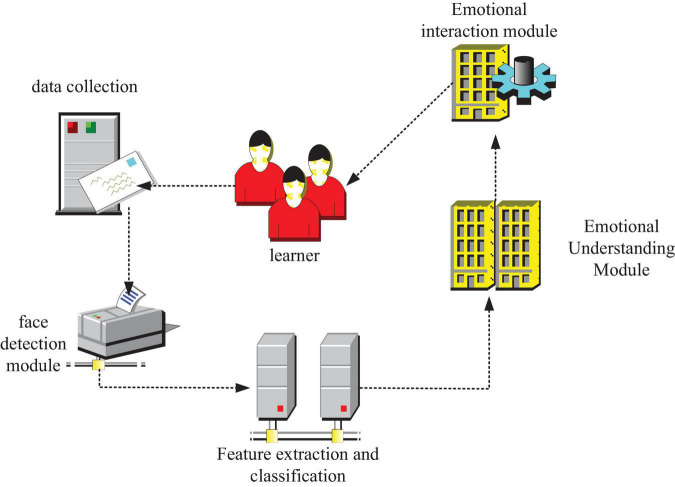
EIMBER overall frame structure.

The EIMBER model takes emotion calculation as the theoretical basis and adopts facial expression recognition as the core technology. By capturing and recognizing the expressions of teenagers, we can judge and understand the emotional state of teenagers, and give corresponding emotional encouragement and encouragement according to the specific content of adolescent health education. In this way, adolescents can be emotionally deficient in health education to a certain extent. The overall frame structure of EIMBER is shown in Figure 6.

As shown in Figure 6, the model consists of five modules: a data acquisition module, a face detection module, a feature extraction and classification module, an emotion understanding module, and an emotion interaction module.

The emotional interaction module automatically retrieves and generates an adjustment plan and an emotional compensation strategy corresponding to the learner’s current emotional state according to the emotional state of the learner automatically understood by the computer. This module is the ultimate goal of EIMBER, which in order to realize the automatic generation of machine emotions and the effect on learners.

**FIGURE 7:**
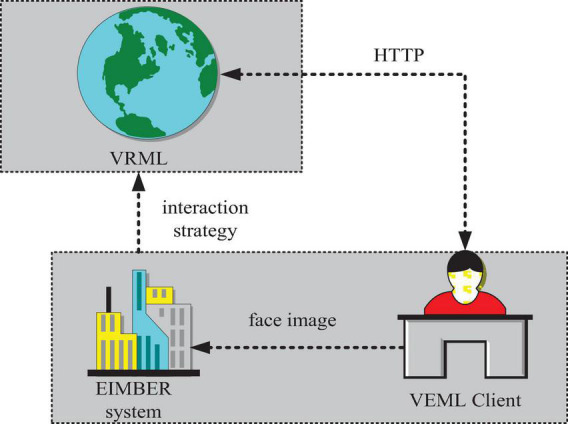
How EIMBER works?

According to the above discussion, EIMBER can realize the recognition function of simple expressions and can generate corresponding adjustment strategies to clear emotions. It helps learners to compensate for the lack of emotion in learning and has the function of promoting human–computer interaction, so it is suitable for application in modern virtual education, and its application principle is relatively simple, as shown in Figure 7.

As shown in Figure 7, EIMBER can work in two ways, one is the front-end, when the program is running, the interface is displayed at the front, and the user needs to manually operate to complete the predetermined workflow. The other is embedded in the background, similar to an Agent or thread. When the program is running, the interface cannot be displayed on the front of the screen, and when no user operation is required, only the user needs to set an automatic running time interval.

The advantage of background embedded is that it runs automatically and does not require the active participation of users, which is very convenient and realistic in the process of health education. Compared with the front-end mode, the naturalness and effectiveness of the emoticon state obtained by the back-end embedded mode will be better. Therefore, the work mode of the back-end embedded mode is mainly used.

## Experiment and analysis of interactive health education model

This manuscript conducts experiments on the constructed interactive health education model. It explores whether the interactive health education model can effectively improve the mental health level of high school students and improve the mental health quality of high school students compared with the traditional manual model. It evaluates the psychological quality of middle school students from three aspects: frustration tolerance ability, social ability, and learning ability.

The experimental subjects of this manuscript are 189 junior high school students from a middle school, of whom the ratio of men and women is even, and their basic conditions are shown in Table 3.

**TABLE 3:** Basic information of 189 junior high school students.

Test subject	Type	Number of people	Percentage
Age	13–15	90	47.6%
	15–17	99	52.4%
Gender	Male	95	50.3%
	Female	94	49.7%

As shown in Table 3, among the 189 junior high school students, 90 were aged 13–15, accounting for 47.6%. The number of people aged 15–17 is 99, accounting for 52.5%, which is not much different.

In order to improve the precision of educational experiment results, this experiment was processed to exclude or minimize the influence of non-experimental factors such as student status, teacher status, mental health data content, etc., on the experiment. This manuscript first analyzes the psychological state of these adolescents, as shown in Table 4.

**TABLE 4:** Mental health status of adolescents.

Grade	First grade	Second grade	Third grade
Healthy	11	11	13
Mildly severe	15	17	20
Moderately severe	16	14	16
Severely severe	17	19	20

As shown in Table 4, because the students’ learning anxiety is more serious, they are more sensitive to academic performance and grade ranking. When grades drop or individuals feel unsatisfactory in a single test, they often feel uneasy and doubt their abilities. As a result, they experience an inferiority complex and a sense of failure, and their self-control to control personal emotions and emotions is relatively poor, and they are easily affected by bad emotions.

**FIGURE 8:**
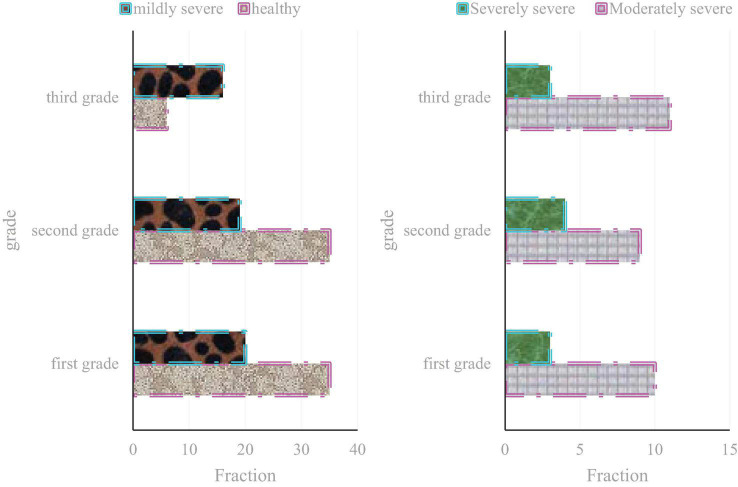
The effect of adopting the interactive health education model.

This manuscript compares and analyzes the mental health education of adolescents after adopting the interactive health education model, as shown in Figure 8.

**FIGURE 9:**
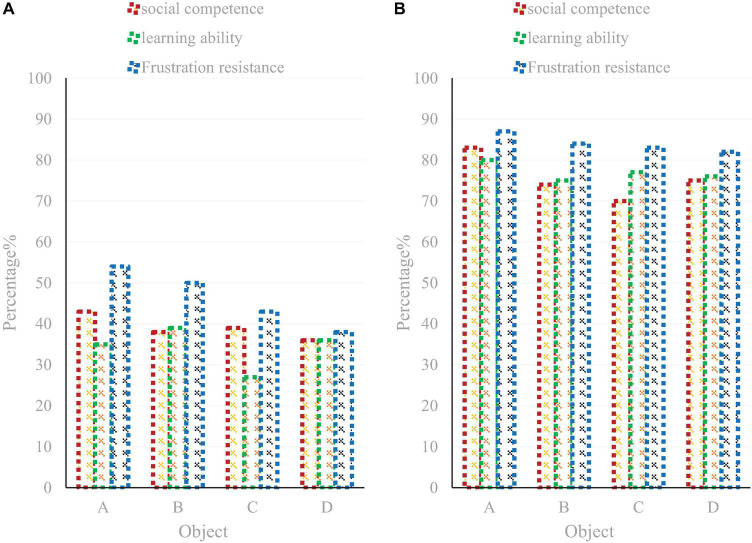
Changes in personality and social ability and learning ability before **(A)** and after **(B)** receiving the interactive health education model.

As shown in Figure 8, interactive health education is a new model of health education with daily education as the basis, classroom education as the main channel, and theme education as an important carrier. With the help of the class teacher and classroom teachers, under the guidance of psychological consultants, with the assistance of parents, and under the attention of all the school staff, their psychological problems have been alleviated to a great extent, they have gotten out of the psychological predicament, and their grades have also been greatly improved. This paper conducts a comparative analysis of the social ability, learning ability, and frustration tolerance of adolescents before and after using the interactive health education model, as shown in Figure 9.

As shown in Figures 9A,B, after receiving the interactive health education model, there are significant differences in the two dimensions of personality and social ability, and in the dimension of learning ability. In the dimension of frustration endurance, although there is no significant difference between the pre- and post-tests, the score of the dimension of frustration endurance has decreased, which indicates that the experimental intervention is effective.

## Conclusion

Adolescents have become an important talent reserve resource in China. Strengthening and improving the health education of college students are of strategic importance to China’s economic and social development and political stability. In the method part, this paper proposes the ICA emotion recognition algorithm and ICABoost algorithm based on emotion calculation and finally found that the ICABoost algorithm has a higher correct rate of emotion recognition. This manuscript proposes an interactive model of the EIMBER system and uses the interactive model of the EIMBER system to identify and classify adolescents’ emotions. In this way, teachers can better grasp the psychological state of students in a timely manner, and when there is an abnormality, they can provide guidance in a timely manner. In order to verify the effectiveness of the interactive health education model proposed in this paper, an experiment was conducted on 189 adolescents in the experimental part, and a comparative analysis was carried out before and after these adolescents received the interactive health education model. It was found that after receiving the interactive health education model, adolescents became more and more mentally healthy, their characters became more cheerful, and their social and learning abilities also improved. The research object of this paper is of great significance, but due to the lack of knowledge of the author, there may be a lot of data errors in the experiment, and the author will definitely do better and better.

## Data availability statement

The original contributions presented in this study are included in the article/supplementary material, further inquiries can be directed to the corresponding author.

## Author contributions

XC: experiment designing and writing. YZ: experiment process implementing. QX: final revision. All authors contributed to the article and approved the submitted version.
